# The value of serum CARDS toxin and NETs formation in children with MPP

**DOI:** 10.3389/fcimb.2026.1750310

**Published:** 2026-03-24

**Authors:** Wanyu Jia, Ruiyang Sun, Jiapu Hou, Zewen Ding, Peng Li, Chunlan Song

**Affiliations:** Henan Province Engineering Research Center of Diagnosis and Treatment of Pediatric Infection and Critical Care, Children’s Hospital Affiliated to Zhengzhou University, Henan Children’s Hospital, Zhengzhou Children’s Hospital, Zhengzhou, Henan, China

**Keywords:** CARDS TX, citrullinated histones 3, mycoplasma pneumoniae pneumonia, myeloperoxidase, neutrophil extracellular traps

## Abstract

**Background:**

Cardiopulmonary Distress Syndrome Toxin (CARDS TX) is a key virulence factor of *Mycoplasma pneumoniae*. Neutrophil extracellular traps (NETs) constitute an important component of innate immunity. This study aims to investigate the changes in CARDS TX and NETs in pediatric severe *Mycoplasma pneumoniae* pneumonia (SMPP) and evaluate their predictive value for SMPP.

**Methods:**

Children hospitalized at Henan Children’s Hospital in 2023 with MPP were enrolled and divided into the GMPP group and SMPP group. Serum levels of CARDS TX, citrullinated histone 3 (CitH3) and myeloperoxidase (MPO), were measured in both groups. Comparative analysis of marker levels between groups was conducted. Diagnostic utility for identifying SMPP was evaluated using logistic regression analysis and receiver operating characteristic ROC curves.

**Results:**

A total of 147 children with mycoplasma pneumonia were included in this study, among which 74 were in the SMPP group and 73 were in the GMPP group. The levels of serum CARDS TX, MPO, and CitH3 in the SMPP group were significantly higher than those in the GMPP group. The levels of serum CARDS TX, MPO, and CitH3 were significantly positively correlated with SMPP, with correlation coefficients of 0.433, 0.357, and 0.396 respectively. The results of multivariate logistic analysis showed that CARDS TX, MPO, and CitH3 were risk factors for SMPP. The AUC of serum CARDS TX, MPO, and CitH3 levels for predicting SMPP were 0.750, 0.706, and 0.729 respectively, with cut-off values of 7.9 pg/ml, 181.16 ng/ml, and 150.38 ng/ml.

**Conclusion:**

The elevated levels of serum CARDS TX, MPO, and CitH3 are independent risk factors for SMPP, and these three indicators have good predictive value for SMPP. CARDS TX secreted by MP may contribute to SMPP development by inducing excessive NET formation.

## 1 Introduction

*Mycoplasma pneumoniae* (MP) is a major pathogen causing community-acquired pneumonia (CAP). Recently the incidence of *Mycoplasma pneumoniae* pneumonia (MPP) has been on the rise, particularly during the 2023-2024, the proportion of MPPs has increased significantly, ranging from 20% to 63% of CAP (Chen et al., 2025; Huang S. et al., 2025; Yan et al., 2025). Moreover, the incidence of macrolide-resistant MP and mixed infections has significantly increased, leading to more severe MPP (SMPP) cases and imposing a substantial burden on children’s health (Centrone et al., 2025; Jiang et al., 2025; Smit et al., 2025). SMPP not only leads to multiple pulmonary and extrapulmonary complications during the acute phase but may also cause structural or functional lung impairment, resulting in long-term complications such as post-infectious obstructive bronchiolitis and bronchiectasis (China NHCotPsRo, 2024). Identifying novel biomarkers for SMPP holds significant importance for early diagnosis and intervention.

The cardiopulmonary distress syndrome toxin (CARDS TX) serves as a key virulence factor of MP, playing a significant role in the progression of MPP (Su et al., 2021). Increased levels of CARDS TX have been observed in both humans and experimental animals during MP infection (Xu et al., 2024). However, the specific mechanism by which CARDS TX promotes the development of MPP remains unclear. The neutrophil extracellular traps (NETs) are a network-like DNA structure composed of de-organized chromatin with pores approximately 200 nanometers in size. Their composition is complex and includes substances such as citrullinated histones 3 (CitH3), granular proteins [such as neutrophil elastase (NE) and myeloperoxidase (MPO)], and so on (Huang et al., 2022). As an important component of innate immunity, neutrophil extracellular traps (NETs) play a role in the development of many diseases, including autoimmune disorders, sepsis, and tumors (Wang et al., 2024). Recent studies have also demonstrated markedly elevated levels of NETs in children with SMPP (Fan and Jiang, 2025), the exact mechanisms underlying NET generation in children with MPP and their pathological roles in disease progression have not been fully elucidated. We speculate that CARDS TX and NETs might jointly be involved in the disease development process of SMPP.

This study aims to investigate the role of CARDS TX and NETs in the occurrence and progression of SMPP in children, and to evaluate their predictive value for SMPP.

## 2 Methods

### 2.1 Patients

This study included pediatric patients hospitalized at Henan Children’s Hospital from September to December 2023 for MPP. Patients were categorized into the mild MPP (GMPP) group and the SMPP group based on disease severity.

The inclusion criteria are as follows: (1) Age between 1 and 18 years, (2) meeting the diagnostic criteria for MPP as outlined in the “Guidelines for Diagnosis and Treatment of *Mycoplasma Pneumoniae* Pneumonia in Children (2023 Edition)” with positive MP DNA or RNA detection in a throat swab, (3) informed consent from the family for participation. Exclusion criteria are as follows: (1) Concurrent infection with other pathogens, (2) children with immune disorders, cardiovascular diseases, connective tissue diseases, or those on long-term corticosteroids or immunosuppressive agents, (3) Children admitted during the convalescent phase of pneumonia.

Grouping criteria are as follows: SMPP group: Meets the diagnostic criteria for SMPP (Seen **Supplementary Materials**). GMPP group: The patient’s condition is relatively mild and does not meet the diagnostic criteria for SMPP.

The sample size of this study was calculated using PASS software. The final calculated sample size was 120 cases, including 60 cases in the GMPP group and 60 cases in the SMPP group.

### 2.2 Data collection

Blood and throat swab samples were collected from all patients within 24 hours of admission for testing. Collect pediatric patient case data, including demographic characteristics, laboratory test results, and imaging examination findings.

Venous Blood Collection: Venous blood samples (1–2 mL) were collected from pediatric patient in the morning under fasting conditions using sterile vacuum tubes without anticoagulants. After collection, the samples were centrifuged at 3000 rpm for 10 minutes. The supernatant (serum) was carefully aspirated using a sterile pipette, aliquoted into sterile EP tubes, and immediately stored at -80 °C for subsequent enzyme-linked immunosorbent assay (ELISA) analysis. All blood collection procedures were performed by trained clinical nurses to ensure sterility and standardization.

Throat Swab Sampling: The patients were instructed to sit upright with their heads tilted back, and the oral cavity was exposed by pressing the tongue down with a tongue depressor. A sterile disposable throat swab was gently inserted into the posterior pharyngeal wall and tonsillar fossa, rotated clockwise and counterclockwise 3–5 times each to fully collect epithelial cells and secretions, avoiding contact with the tongue, teeth, and oral mucosa to prevent sample contamination. After sampling, the swab was quickly inserted into a sterile sample collection tube containing 1 ml of sterile normal saline, broken at the marked position, and the tube cap was tightly closed. The sample tubes were shaken vigorously for 1 minute to fully elute the collected substances, and then stored at 4 °C and transported to the laboratory within 2 hours for subsequent processing.

Detect levels of peripheral blood CARDS TX (mlbio, Shanghai), CitH3 (Wuhan Fine Biotech Co., Ltd., Wuhan), and MPO (Elabscience Biotechnology Co., Ltd., Wuhan) using the enzyme-linked immunosorbent assay (ELISA) method. The absorbance at 450 nm was measured within 15 minutes using an ELISA reader (Bio-Rad, Hercules, USA), and the concentration of each target indicator was calculated using the standard curve. Specific operational procedures followed the kit instructions.

### 2.3 Statistical analyses

Data analysis was performed using SPSS 26.0. The Kolmogoro v-Smirnov test was used to assess normality for continuous variables. Variables meeting normality requirements were expressed as mean ± standard deviation, while those not meeting normality were expressed as median (interquartile range). The t-test or Mann-Whitney U test was used to compare clinical characteristics and levels of CARDS TX, MPO, and CitH3 between groups. Spearman correlation analysis assessed the relationship between CARDS TX, MPO, CitH3 levels, and SMPP. Multivariate logistic regression analysis evaluated risk factors for SMPP. The receiver operating characteristic (ROC) curve assessed the diagnostic value of these indicators for SMPP. P < 0.05 indicates that the difference is statistically significant.

## 3 Results

### 3.1 Clinical characteristics and levels of CARDS TX, MPO, CitH3

**Figure 1:**
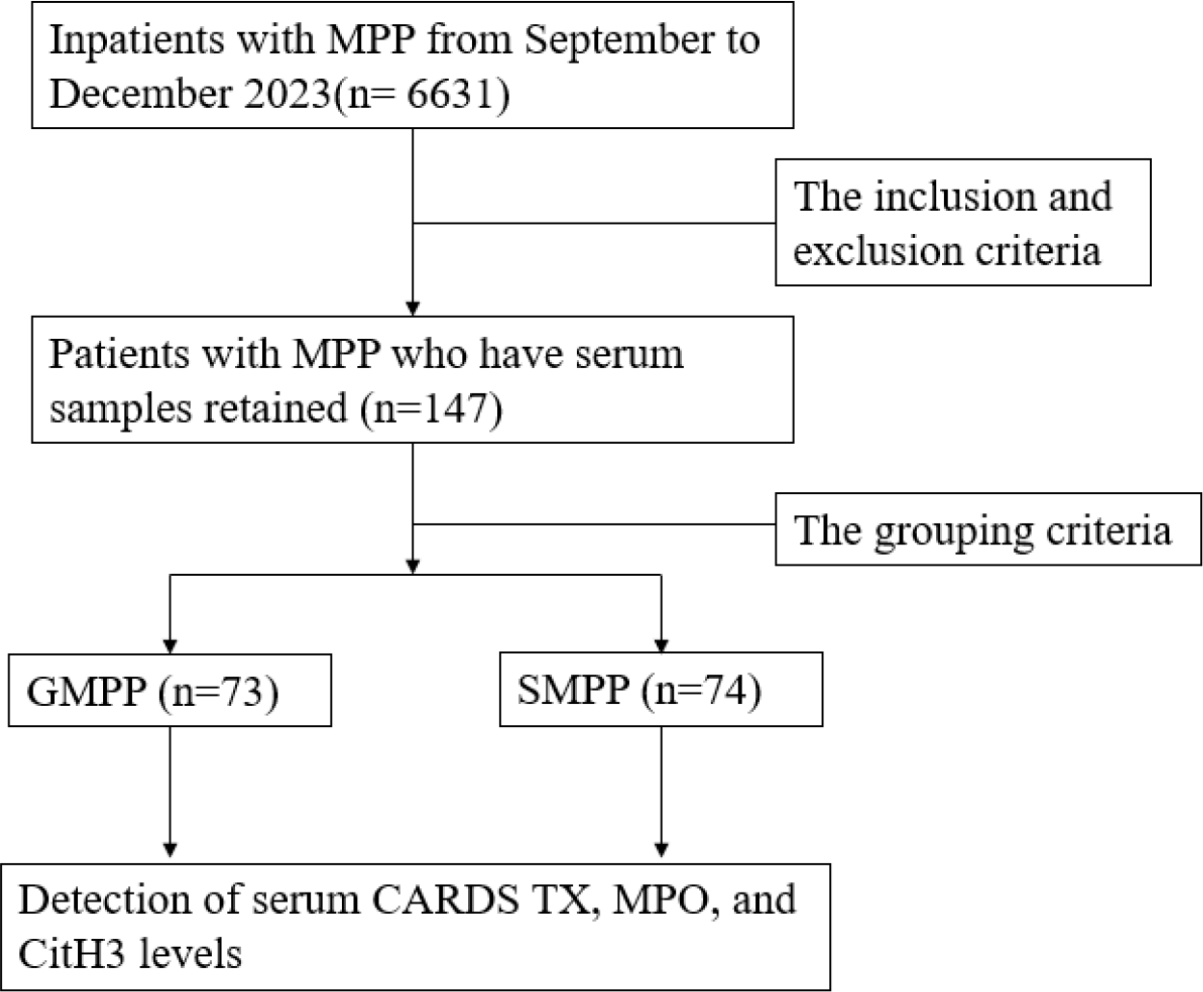
Flow diagram.

**Figure 2:**
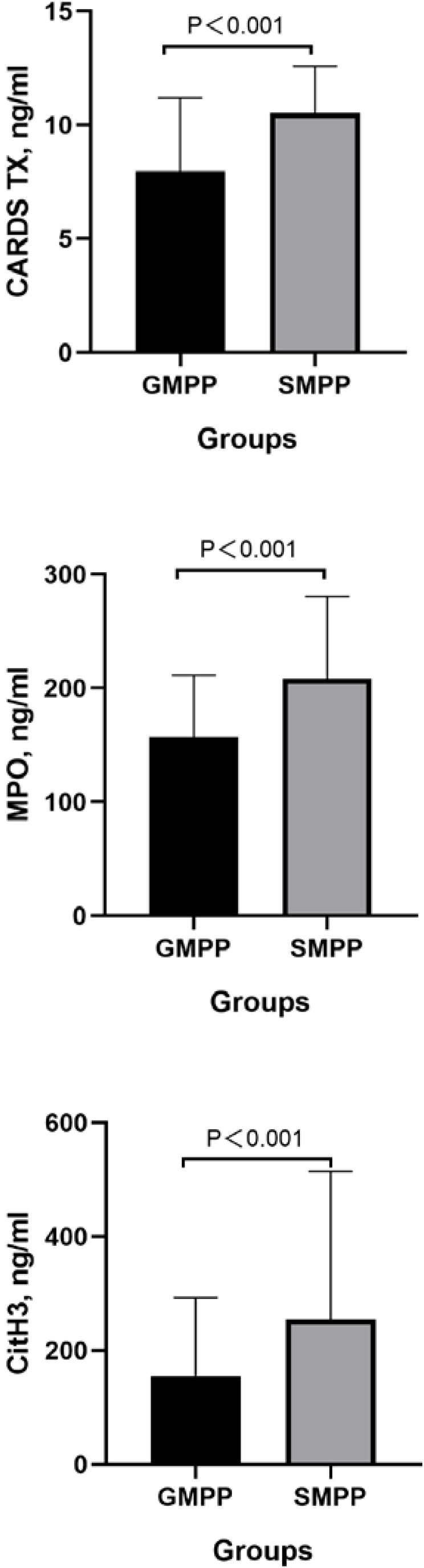
Comparison of CARDS TX, MPO, and CitH3.

This study included 147 patients with MPP, comprising 81 males and 66 females, with a mean age of 7.25 ± 0.23 years.The flowchart is shown in **Figure 1**. The flowchart is shown in **Figure 1**. The SMPP group consists of 74 patients. No significant differences were observed between the two groups in terms of age or gender (P > 0.05). The duration of fever in the SMPP group was significantly longer than that in the GMPP group. And the length of stay for patients in the SMPP group was significantly longer. (P<0.05) There was no statistically significant difference in white blood cell (WBC) count or neutrophil count between the two groups (P > 0.05). Serum inflammatory markers neutrophil-to-lymphocyte ratio (NLR), C-reactive protein (CRP), procalcitonin (PCT), and Interleukin-6 (IL-6) were significantly higher in the SMPP group, with statistically significant differences. Serum levels of CARDS TX, MPO, and CitH3 were also significantly higher in the SMPP group, seen in **Table 1**, **Figure 2**.

**Table 1:** Comparison of data between the GMPP group and the SMPP group.

	SMPP (n=74)	GMPP (n=73)	*p*
Gender, Male/Female	41/33	40/33	0.941
Age, years	7.20 ± 0.27	7.30 ± 0.37	0.821
length of stay, days	9.00 (8.00,11.00)	7.00 (6.00,8.00)	<0.001
duration of fever, days	8.00 (6.00, 11.00)	5.00 (3.50, 7.00)	<0.001
WBC, 10^9/L	8.34 (5.95, 11.26)	8.21 (6.26, 11.33)	0.583
neutrophil, 10^9/L	5.17 (3.93, 7.79)	4.68 (3.35,7.66)	0.673
NLR	2.71 (1.64, 4.40)	2.00 (1.14, 2.88)	0.005
CRP, mg/L	11.25 (2.80, 23.06)	5.44 (1.83, 14.17)	0.034
PCT, mg/L	0.097 (0.056, 0.17)	0.063 (0.039, 0.084)	<0.001
IL-6, mg/L	10.04 (5.75, 33.08)	7.35 (2.54, 14.17)	0.006
CARDS TX (pg/ml)	9.87 (9, 11.85)	7.90 (5.39, 10.35)	<0.001
MPO (ng/ml)	208.25 ± 8.41	157.42 ± 6.28	<0.001
CitH3 (ng/ml)	198.93 (159.39, 252.83)	137.99 (77.32, 214.68)	<0.001

### 3.2 Correlation analysis

According to Spearman’s correlation analysis, serum levels of CARDS TX, MPO, and CitH3 showed significant positive correlations with SMPP, with correlation coefficients of 0.433, 0.357, and 0.396, respectively. Serum MPO and CitH3 levels also correlated positively with CARDS TX, yielding correlation coefficients of 0.257 and 0.288, respectively (seen in **Table 2**).

**Table 2:** Spearman correlation coefficients between the three indicators and SMPP.

Variables	SMPP	CARDS	MPO	CitH3
SMPP	1.000	.433**	.357**	.396**
CARDS TX	.433**	1.000	.257**	.288**
MPO	.357**	.257**	1.000	.021
CitH3	.396**	.288**	.021	1.000

^**^At the 0.01 level (two-tailed), the correlation is significant.

### 3.3 Risk factors for SMPP

Univariate logistic regression analysis results suggest that NLR, PCT, CARDS TX, MPO, and CitH3 are potential risk factors for SMPP. Multivariate logistic regression analysis revealed that CARDS TX, MPO, and CitH3 are risk factors for SMPP, with odds ratios (OR) of 1.309 (1.104, 1.552), 1.015 (1.007, 1.023), and 1.006 (1.001, 1.011), respectively (**Table 3**).

**Table 3:** Univariate and multivariate logistic analyses of factors affecting SMPP.

Variables	Univariate logistic analysis			Multivariate logistic analysis		
	*P*	OR	95%CI	*P*	OR	95%CI
NLR	0.014	1.258	1.047-1.510			
PCT	0.034	30.749	1.290-732.778			
CARDS TX	<0.001	1.445	1.239-1.685	0.002	1.309	1.104-1.552
MPO	<0.001	1.015	1.008-1.022	<0.001	1.015	1.007-1.023
CitH3	0.002	1.007	1.002-1.011	0.015	1.006	1.001-1.011

### 3.4 ROC curves for SMPP

**Figure 3:**
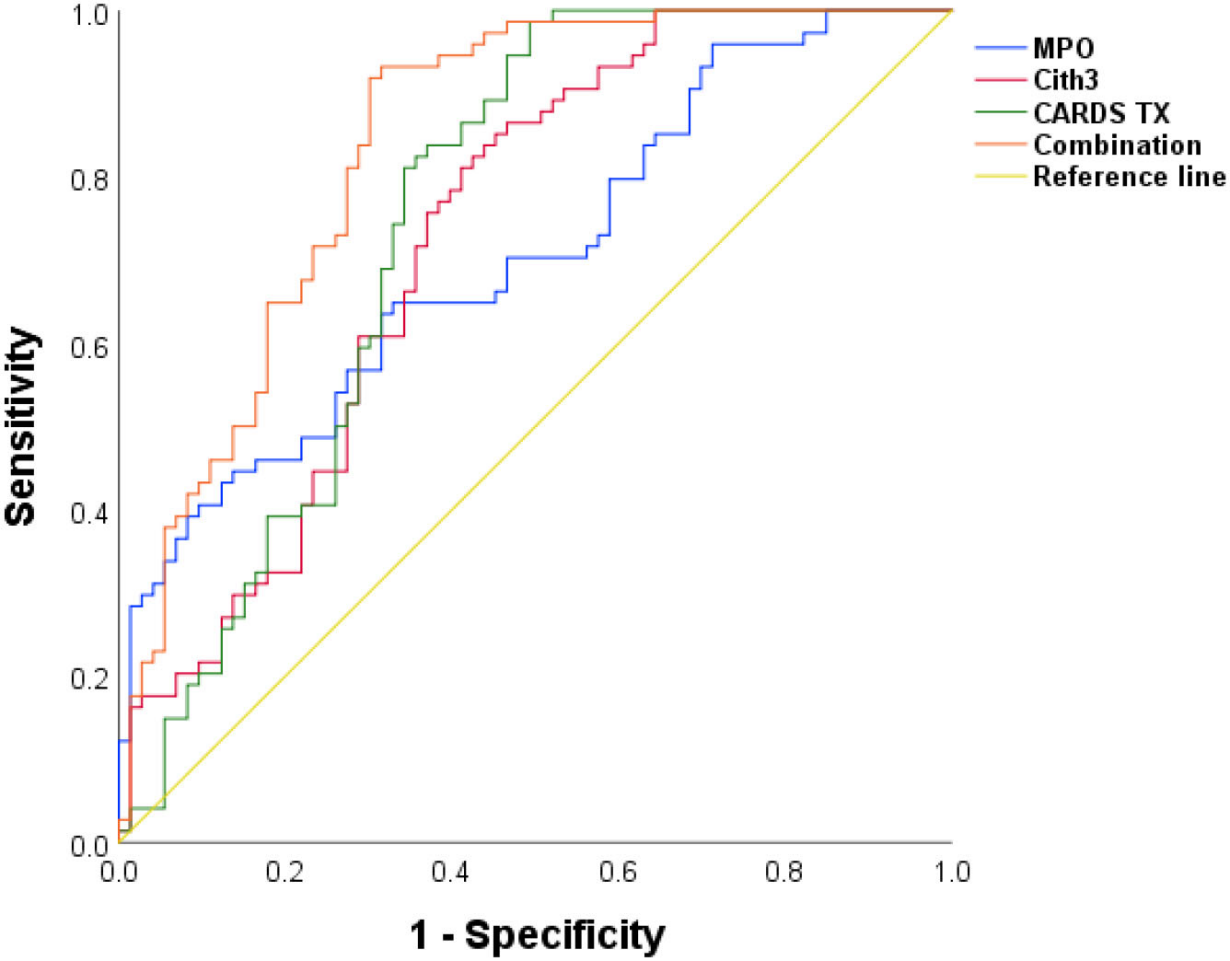
ROC curves for serum CARDS TX, MPO, CitH3, and their combined prediction of SMPP.

The ROC analysis indicated that the AUC values for predicting SMPP using serum CARDS TX, MPO, and CitH3 levels were 0.750, 0.706, and 0.729, respectively. The cutoff values for serum CARDS TX, MPO, and CitH3 are 7.9 pg/mL, 181.16 ng/mL, and 150.38 ng/mL. The combined AUC for predicting SMPP using these three markers is 0.840 (0.770, 0.895), which is higher than that of any single marker, while sensitivity and specificity also increase. (**Table 4**, **Figure 3**).

**Table 4:** ROC results of serum CARDS TX, MPO, CitH3, and their combined use for predicting SMPP.

Variables	AUC	Sensitivity, %	Specificity, %	Cut-off value	Youden index
CARDS TX	0.750 (0.672,0.818)	98.65	50.68	7.9	0.4933
MPO	0.706 (0.625,0.778)	63.51	68.49	181.16	0.3201
CitH3	0.729 (0.649,0.799)	81.08	58.90	150.38	0.3999
Combination	0.840 (0.770,0.895)	91.89	69.86	/	0.6175

## 4 Discussion

MPP is a common community-acquired pneumonia in children. Most cases present with mild symptoms and have a favorable prognosis, but some patients may progress to SMPP (Zhang et al., 2024). In recent years, due to the rising rate of MP resistance, as well as factors such as mixed infections, the incidence of SMPP has also increased (Huang and Shi, 2025; Yan et al., 2025). This study analyzed the serum samples of 147 children and found that the levels of CARDS TX, MPO, and CitH3 in children with SMPP were significantly elevated. Moreover, the multivariate logistic regression analysis indicated that these three indicators were risk factors for SMPP, providing new biological markers for the diagnosis of SMPP.

Community-acquired respiratory distress syndrome toxin (CARDS TX), initially identified and named by Kannan (Kannan et al., 2005), is a protein specific to MP that binds to human surfactant protein A. It plays a crucial role in the pathogenesis of diseases associated with MP infection (Fang et al., 2025). Previous studies have found elevated levels of CARDS toxin expression in the bronchoalveolar lavage fluid (BALF) of children with MPP, particularly when MPP is complicated by mucus plugs and pleural effusion (Wang et al., 2022). In mice infected with MP via the intranasal route, BALF levels of CARDS TX correlated with MP cell burden and the severity of lung tissue inflammation, suggesting that higher MP counts, elevated CARDS TX levels, and more severe disease progression (Su et al., 2021). Our study also found that the level of CARDS TX in the serum of children with SMPP was significantly elevated and showed a significant positive correlation with SMPP. Moreover, CARDS TX is an independent risk factor for SMPP, further confirming that elevated levels of CARDS TX are involved in the progression of SMPP. Previous studies have shown that the CARDS TX can trigger excessive immune inflammatory responses, thereby exacerbating the MPP condition (Wang et al., 2022). CARDS TX enhances NLRP3 inflammasome activation, thereby participating in multiple signaling pathways that cause cellular damage (Song et al., 2024). Studies have also confirmed that CARDS TX relies on the TLR2/CD14/MyD88 pathway to promote HMGB1 release and contributes to the progression of MPP disease (Fan et al., 2022). It should be noted that our results indicate that the CARDS TX test alone has high sensitivity but low specificity. Therefore, it may be more suitable for early high-sensitivity screening. However, for the definitive diagnosis of SMPP, it still needs to be determined through a comprehensive assessment of other indicators. Our study revealed that the levels of serum MPO and CitH3 in children with MPP were positively correlated with the level of CARDS TX. MPO and CitH3 are important components of NETs. Therefore, we supposed that CARDS TX and NET jointly participate in the progression of MPP disease, exacerbating the body’s inflammatory response.

In 2004, Brinkmann et al. first discovered that activated neutrophils can participate in capturing and killing pathogens by releasing NETs (Brinkmann et al., 2004). In recent years, in-depth research on NETs has revealed that these structures play a double-edged role in infections caused by various pathogens. When NETs are overproduced or not cleared promptly, their abnormal overaccumulation exacerbates further damage to local tissues and organs, contributing to the pathogenesis of multiple diseases (Adrover et al., 2023; Zhang et al., 2023). Research has demonstrated that serum NET levels correlate significantly with the severity of sepsis, and it has been found that NETs promote the development of sepsis-associated acute lung injury (Zou et al., 2023). Compared to healthy controls, higher levels of NETs were detected in the serum of COVID-19 patients. Similarly, substantial amounts of NETs were found in tracheal aspirates and lung tissue from critically ill COVID-19 patients, significantly exceeding levels from healthy controls (Veras et al., 2020). Our study also found that MPO and CitH3, key components of NETs, were significantly elevated in children with SMPP and positively correlated with SMPP severity, indicating they are independent risk factors for SMPP. This suggests that NETs are similarly activated in children with MPP and contribute to disease progression. Xia et al. similarly found extensive NETs in blood, BALF, and tissue samples from children with MPP, which correlated with disease severity, consistent with the findings of our study (Huang X. et al., 2025).Previous studies have shown that NETs can be induced by various substances, including endogenous products within the body and molecular signals from pathogens (Speziale and Pietrocola, 2021; Hidalgo et al., 2022). Our research has found that the concentration of CARDS TX is positively correlated with MPO and CitH3. Therefore, we speculate that CARDS TX might activate NETs, thereby triggering a series of inflammatory responses in the body and exacerbating the lung damage in children with MPP.

This study has several limitations. As a single-center investigation with a relatively small sample size, future research should expand the sample size and conduct multicenter studies to further validate these findings. The study only measured serum CARDS TX and NETs levels in pediatric patients without exploring underlying mechanisms, though it lays the groundwork for subsequent research. We will now conduct in-depth investigations into the specific mechanisms by which CARDS TX induces NET formation.

## 5 Conclusion

Elevated serum levels of CARDS TX, MPO, and CitH3 are independent risk factors for SMPP. All three markers demonstrate good predictive value for SMPP, and their combined use enhances predictive accuracy. CARDS TX secreted by MP may contribute to SMPP development by inducing excessive NET formation.

## Data Availability

The original contributions presented in the study are included in the article/**Supplementary Material**. Further inquiries can be directed to the corresponding author.
