# Supplementary material for: The value of serum CARDS toxin and NETs formation in children with MPP
Source: Front Cell Infect Microbiol. 2026 Mar 24;16:1750310. doi: 10.3389/fcimb.2026.1750310 (PMC13079613; doi:10.3389/fcimb.2026.1750310)
Supplement: Supplementary file 1 [file Table1.docx]

Diagnostic Criteria for SMPP: Meeting any one of the following manifestations:

1. Persistent high fever (≥39°C) for ≥5 days or fever for ≥7 days, with no decrease or even an increase in peak body temperature, resulting in high fever;

2. Presence of one or more symptoms: wheezing, shortness of breath, dyspnea, chest pain, or hemoptysis. These manifestations correlate with severe disease, coexisting plastic bronchitis, asthma attacks, pleural effusion, or pulmonary embolism;

3. Development of extrapulmonary complications;

4. Resting oxygen saturation ≤0.93% at sea level breathing room air;

5. Imaging findings meeting any of the following:

(1) High-density consolidation involving approximately 2/3 or more of a single lung lobe, or high-density consolidation in two or more lung lobes (regardless of extent);

(2) Unilateral or bilateral diffuse bronchiolitis, possibly with bronchitis (thickened bronchial walls, peribronchial exudate, and possible mucus plug obstruction causing atelectasis) .

6. Progressive worsening of clinical symptoms with imaging showing disease progression exceeding 50% within 24-48 hours;

7. Significant elevation in any one of CRP, LDH, or D-dimer.
